# Molecular Simulations of Carbohydrates with a Fucose-Binding *Burkholderia ambifaria* Lectin Suggest Modulation by Surface Residues Outside the Fucose-Binding Pocket

**DOI:** 10.3389/fphar.2017.00393

**Published:** 2017-06-21

**Authors:** Tamir Dingjan, Anne Imberty, Serge Pérez, Elizabeth Yuriev, Paul A. Ramsland

**Affiliations:** ^1^Medicinal Chemistry, Monash Institute of Pharmaceutical Sciences, Monash UniversityMelbourne, VIC, Australia; ^2^Centre de Recherches sur les Macromolécules Végétales, Centre National de la Recherche Scientifique UPR5301, Université Grenoble AlpesGrenoble, France; ^3^Département de Pharmacochimie Moléculaire, Centre National de la Recherche Scientifique, UMR5063, Université Grenoble AlpesGrenoble, France; ^4^School of Science, RMIT UniversityMelbourne, VIC, Australia; ^5^Department of Surgery Austin Health, University of MelbourneMelbourne, VIC, Australia; ^6^Department of Immunology, Central Clinical School, Monash UniversityMelbourne, VIC, Australia; ^7^Burnet InstituteMelbourne, VIC, Australia

**Keywords:** blood group determinants, *Burkholderia ambifaria*, docking, fucose, molecular dynamics

## Abstract

*Burkholderia ambifaria* is an opportunistic respiratory pathogen belonging to the *Burkholderia cepacia* complex, a collection of species responsible for the rapidly fatal cepacia syndrome in cystic fibrosis patients. A fucose-binding lectin identified in the *B. ambifaria* genome, BambL, is able to adhere to lung tissue, and may play a role in respiratory infection. X-ray crystallography has revealed the bound complex structures for four fucosylated human blood group epitopes (blood group B, H type 1, H type 2, and Le^x^ determinants). The present study employed computational approaches, including docking and molecular dynamics (MD), to extend the structural analysis of BambL-oligosaccharide complexes to include four additional blood group saccharides (A, Le^a^, Le^b^, and Le^y^) and a library of blood-group-related carbohydrates. Carbohydrate recognition is dominated by interactions with fucose via a hydrogen-bonding network involving Arg15, Glu26, Ala38, and Trp79 and a stacking interaction with Trp74. Additional hydrogen bonds to non-fucose residues are formed with Asp30, Tyr35, Thr36, and Trp74. BambL recognition is dominated by interactions with fucose, but also features interactions with other parts of the ligands that may modulate specificity or affinity. The detailed computational characterization of the BambL carbohydrate-binding site provides guidelines for the future design of lectin inhibitors.

## Introduction

Cystic fibrosis morbidity is mostly due to respiratory infection by opportunistic pathogens (Lyczak et al., 2002; O'Sullivan and Freedman, 2009; Ciofu et al., 2013; Caverly et al., 2015). *Burkholderia cepacia* is one of the most dangerous pathogens isolated from cystic fibrosis patients; 20% of infected individuals succumb to a rapidly fatal pneumonia termed “cepacia syndrome” (Zahariadis et al., 2003; Blackburn et al., 2004; Lynch, 2009). Isolated *B. cepacia* strains have been classified into a steadily increasing number of species, referred to collectively as the *B. cepacia* complex (currently consisting of 20 species Vandamme et al., 1997; De Smet et al., 2015; Martinucci et al., 2016). Most members of the complex are resistant to multiple clinically used antibiotics, making the search for new therapeutics more urgent (Zhou et al., 2007; Loutet and Valvano, 2011; Podnecky et al., 2015). *Burkholderia ambifaria*, a member of the *B. cepacia* complex, has been isolated from both clinical and environmental samples (Coenye et al., 2001). In addition to infecting human respiratory tissue, *B. ambifaria* can colonize plant rhizospheres, where it promotes growth and protects against invading fungi (Li et al., 2002; Lee et al., 2006; Parra-Cota et al., 2014).

Previously, a carbohydrate-binding protein (named “BambL”) was identified in the *B. ambifaria* genome; binding studies using human tissues suggest it may play a role in infection (Audfray et al., 2012). Opportunistic bacteria often adhere to tissues by binding to host carbohydrates using carbohydrate-recognizing proteins (lectins) displayed at the bacterial surface (Bavington and Page, 2005; Imberty and Varrot, 2008; Pieters, 2011; Audfray et al., 2013). Among the many carbohydrates present on human cells, fucose-bearing blood group determinants are often recognized by bacterial lectins (Lindén et al., 2008; Anstee, 2010; Holmner et al., 2010). In the cystic fibrosis respiratory epithelium, cell-surface carbohydrates, present on glycolipids, N-glycoproteins, and mucins, are more fucosylated than in healthy tissue (Rhim et al., 2001; Venkatakrishnan et al., 2015). This increased fucosylation may promote adhesion by fucose-recognizing pathogens (Stoykova and Scanlin, 2008; Audfray et al., 2013). Known cystic fibrosis pathogens *Pseudomonas aeruginosa, Burkholderia cenocepacia* and *Aspergillus fumigatus*, all have lectins that bind to fucosylated human blood group carbohydrates (Mitchell et al., 2002; Imberty et al., 2004; Sulak et al., 2010, 2011; Houser et al., 2013, 2015). Significantly, the *P. aeruginosa* lectins are strongly associated with respiratory tissue damage and bacterial load in a mouse model of lung injury, and treatment with monosaccharides, able to specifically inhibit lectin binding, reduces infection (Chemani et al., 2009). Similar effects have been reported in a human *P. aeruginosa* infection case study (von Bismarck et al., 2001) suggesting that interfering with lectin-carbohydrate interactions may offer a new frontier in anti-infective treatment (Sharon, 2006; Pera and Peters, 2014). Lectin inhibitor design begins with a thorough understanding of the role of each functional group in the natively recognized carbohydrate (Ernst and Magnani, 2009).

**Figure 1:**
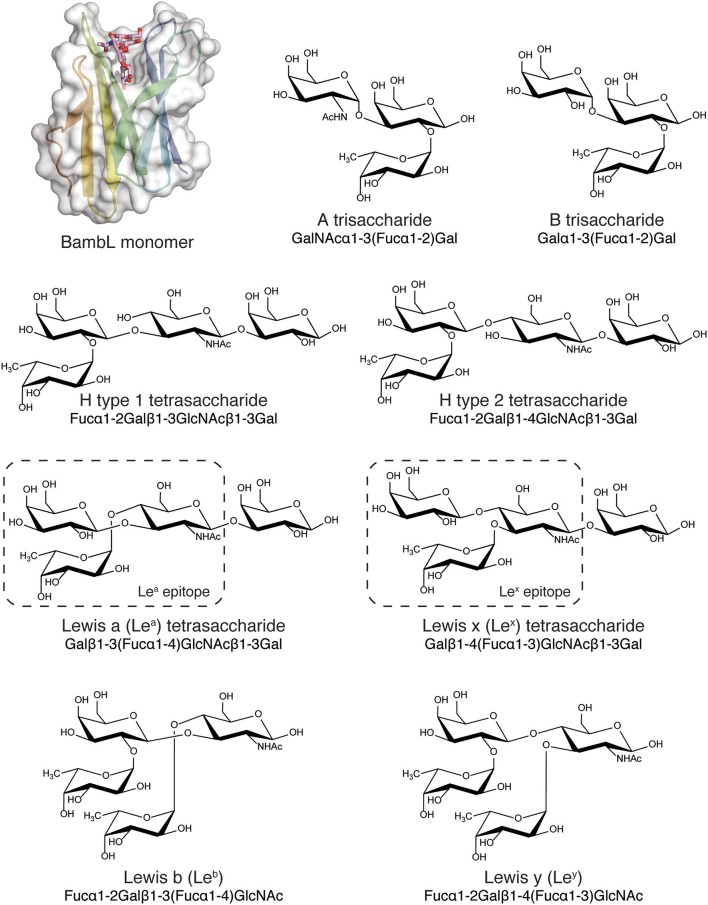
BambL subunit shown with blood group and tissue antigen saccharides (A, B, H, Le^a^, Le^b^, Le^x^, and Le^y^) used for simulation. BambL structure from PDB ID: 3ZZV, with the intra-protomeric binding site and ligand shown.

The crystallographic structure of BambL has been solved, revealing a six-bladed β-propeller fold formed by three separate protomers (Audfray et al., 2012). Each subunit contains a single carbohydrate-binding site; upon oligomerization, three additional binding sites are formed at the interfaces between protomers, for a total of six binding sites in the β-propeller fold. The intra- and inter-protomeric sites have similar architectures and (for most blood group carbohydrates) similar binding properties. For this reason, the present work addresses interactions within the intra-protomeric site only. Crystal structures of BambL have also been obtained bound to multiple fucosylated human blood group tetrasaccharides: H type 1, H type 2, B type 2, and Le^x^ (PDB IDs: 3ZW2, 3ZZV, 3ZWE, and 3ZW1; Audfray et al., 2012; Topin et al., 2013; Figure 1). In each case, the carbohydrate is bound via a buried fucose residue, which participates in a network of hydrogen bonds within a tight fucose-binding pocket. Blood group carbohydrate binding specificity has also been determined by glycan array and affinity quantified by titration microcalorimetry: strongest affinity is for H type 2 tetrasaccharide (*K*_*D*_ 7.5 μM) and Le^y^ pentasaccharide (*K*_*D*_ 11.1 μM; Audfray et al., 2012). This binding preference indicates that BambL is more selective for blood and tissue carbohydrate determinants containing the type 2 epitope Fucα1-2Galβ1-4GlcNAc. Several of the blood group and tissue antigens recognized by BambL have not been structurally characterized in complex with the lectin (e.g., Le^y^, Le^b^, and A). Additionally, while existing crystal structures describe static recognition, the dynamic behavior of BambL complexes has not been described. The relative contributions of individual binding interactions to saccharide recognition is also unknown. Extending the structural analysis of BambL-blood group complexes to probe these aspects of recognition will enhance understanding of carbohydrate recognition and facilitate inhibitor design.

The goal of this computational study was to characterize BambL-saccharide binding modes and to inform future *in silico* or structure-based design of inhibitors for this bacterial lectin. We were interested in identifying lectin residues that are critical for ligand recognition and thus could be used as constraints in prospective virtual screening. In particular, we investigated whether the BambL binding site is restricted to recognizing fucose or is capable of engaging non-fucose saccharides using additional interactions. We first used docking and site mapping to study binding modes in complexes featuring A, B, O (H), and Lewis fucosylated carbohydrates and a library of blood-group-related saccharides. The dynamic behavior of these systems was then explored by molecular dynamics (MD) simulations. The recognition of fucose-containing saccharides by BambL is accomplished by a hydrogen-bonding network between fucose and Arg15, Glu26, Trp79, and to a lesser extent Ala38. A hydrophobic contact is made between the fucose non-polar face and the Trp79 imidazole. Additional hydrogen bonds outside the fucose-binding pocket to Asp30, Thr36, Trp74, and Tyr35 are formed in complex with multiple blood group and blood-group-related saccharides. Residues involved in these interactions are consistently engaged by blood-group-related saccharides, suggesting they may be valuable interaction targets for BambL inhibitors.

## Materials and methods

A single BambL subunit containing an intra-protomeric (Audfray et al., 2012) binding site was used in the below computational studies.

### Blood-group and blood-group-related carbohydrate structure generation

Low energy blood-group and blood-group-related carbohydrate structures were generated and simulation parameters produced using the GLYCAM web portal (Woods, 2005; Kirschner et al., 2008). The A and B determinants were modeled as trisaccharides for comparison to previous binding data for the soluble type A determinant (Audfray et al., 2012). The H type 1, H type 2, Le^a^ and Le^x^ determinants were modeled as tetrasaccharides for consistency to previously determined binding data (Audfray et al., 2012) and the Le^b^ and Le^y^ determinants were modeled as tetrasaccharides to encompass the entire epitope. The library of blood-group-related structures is shown in Supplementary Figure 1.

### Docking

Docking experiments were performed using the docking program Glide 6.8 (Friesner et al., 2004, 2006; Halgren et al., 2004; Schrödinger, 2014a) available within the molecular modeling package Maestro (Schrödinger, 2014a,b). The BambL crystallographic complexes were downloaded from the Protein Data Bank, PDB (Berman et al., 2000), and the protein structures prepared using the Protein Preparation Wizard tool (Madhavi Sastry et al., 2013; Schrödinger, 2014b). During this step, structural details required for the docking calculation were specified. Double bond orders were applied for backbone carbonyl and aromatic side chain moieties, hydrogen atoms were added to the structure, water molecules removed, and disulfide bonds created between cysteine side chain sulfur atoms in close proximity. Missing atoms and side chains were added based on the protein's primary sequence using the Prime tool (Schrödinger, 2014c). To remove steric clashes between added hydrogen atoms, a minimization step was then conducted on hydrogen atoms only, using the OPLS2005 forcefield (Banks et al., 2005). A receptor grid was generated using default settings, with the binding site box centered on the crystallographic ligand. Ligands were docked into the receptor grid using Standard Precision mode with default settings. All carbohydrate atoms were treated flexibly during docking, including all glycosidic linkages and exocyclic groups. The lowest-energy docked poses were retained for MD simulation. Docked poses were filtered by glycosidic dihedral angle to exclude unfavorable high energy carbohydrate conformations. Cutoff values for dihedral filtering were chosen for each glycosidic linkage based on isoenergy contours previously calculated with the MM3 force field from Imberty et al. (1995). Conformations with dihedrals in the following ranges were removed from the analysis: Fucα1-2Gal φ < −130° & 180° < ψ <360°; GalNAcα1-3Gal φ > 240°; Galβ1-3GlcNAc φ > 0° & 180° < ψ < −60°. Thus, we have used energy maps to post-filter docked poses as a means of retaining reasonable conformations. These energy maps have been commonly used to evaluate carbohydrate conformations obtained from simulations and experimental work [for example Jackson et al. (2014) and Tempel et al. (2002)]. Hydrogen bonds and contacts were tallied using MDAnalysis (Michaud-Agrawal et al., 2011; distance = 3.0 Å, angle = 120).

### Site mapping

All BambL-blood group carbohydrate complexes were examined using LigPlot (Wallace et al., 1995; Laskowski and Swindells, 2011). Only poses that passed the glycosidic torsion filter requirements (see above), were used for site mapping, following a previously developed method (Yuriev et al., 2001; Agostino et al., 2009b, 2011, 2013; Dingjan et al., 2015a). In brief, each individual hydrogen bond made by a particular BambL residue was counted toward the hydrogen-bond tally. Non-polar vdW interactions between a specific BambL residue and a carbohydrate residue were counted as a single interaction toward the tally. The tallies were normalized to percentages of the total number of hydrogen bond or vdW interactions. Site maps were generated using residue inclusion cutoff values for lectin-carbohydrate complexes of 90% for hydrogen bonds, 0% for vdW interactions (Agostino et al., 2013). Site map images were rendered using PyMOL (Schrödinger, 2014d).

### Molecular dynamics

MD simulations were performed using Gromacs 5.0.4 (Berendsen et al., 1995; Van Der Spoel et al., 2005; Hess et al., 2008; Pronk et al., 2013). Proteins were parameterized using the AMBER99SB-ILDN (Lindorff-Larsen et al., 2010) forcefield. Carbohydrate topologies were generated using the GLYCAM06 (Kirschner et al., 2008) force field via the glycam.org web portal. The resulting AMBER-formatted topology was converted to GROMACS format using the “acpype” tool (Sousa da Silva and Vranken, 2012). The correctly formatted carbohydrate topology was then combined with the protein topology to describe the entire protein-carbohydrate system. Protein-carbohydrate docked complexes were placed in a rhombic dodecahedral box with a 10 Å minimum distance between solute and box wall, and subsequently solvated using the TIP3P water model. To maintain electrostatic neutrality, Na^+^ and Cl^−^ counterions were added by the *genion* module. To remove steric clashes between nearby atoms, the system contents were minimized using the steepest descent algorithm (maximum steps: 50,000). The positions and velocities of the solvent molecules and ions were then equilibrated at constant volume and temperature (NVT ensemble) using three restraint settings: with all protein heavy atoms restrained for 100 ps, then with only backbone atoms restrained for 100 ps (both at 10 K), followed by a 100 ps equilibration without restraints at 300 K. Finally, the pressure of the system was equilibrated for 300 ps without restraints at constant atmospheric pressure (NPT ensemble) at 310 K. During all equilibration steps, positional restraints were applied to protein residues using LINCS (Hess, 2007). The coordinates from the final equilibration step were used to begin production simulation, which was conducted for 400 ns.

For all MD simulations in the NPT ensemble, temperature was kept constant using the velocity rescaling thermostat coupled with a time constant of 0.1 ps. Pressure was held constant at 1 bar using the Parrinello-Rahman barometer, coupled with a time constant of 2 ps. Equations of motion were integrated using a leap-frog integrator with a 2 fs timestep. Long-range electrostatics were evaluated using the Particle Mesh Ewald method. Cutoff values for Coulomb and vdW interactions were set to 1.0 nm. Complexes with blood group carbohydrate ligands were simulated in triplicate, complexes with blood-group-related carbohydrate ligands were simulated in singlicate. Each replicate was commenced using randomized velocities, resulting in independent simulations with different initial velocities.

#### Analysis of MD simulations

Hydrogen bonds in MD simulations were analyzed using the Baker-Hubbard method implemented in the MDTraj (McGibbon et al., 2015) software library. An occupancy value was assigned to each hydrogen bond by calculating the percentage of simulation frames in which the bond was present. Glycosidic dihedral angles were measured using MDTraj and compared to calculated isoenergy contours (see above). Carbohydrate ring conformations were analyzed using Best Four-Member Plane method from GLYCAM (Makeneni et al., 2014). CH-π interactions were represented by measuring a shortest distance from either of the fucose atoms C3, C4, C5, or C6 to atoms of the indole ring of Trp74. Atom labeling corresponds to the conventions of the PDB exchange dictionary (Berman et al., 2003).

## Results

### Generation of BambL-blood group complexes by docking

**Figure 2:**
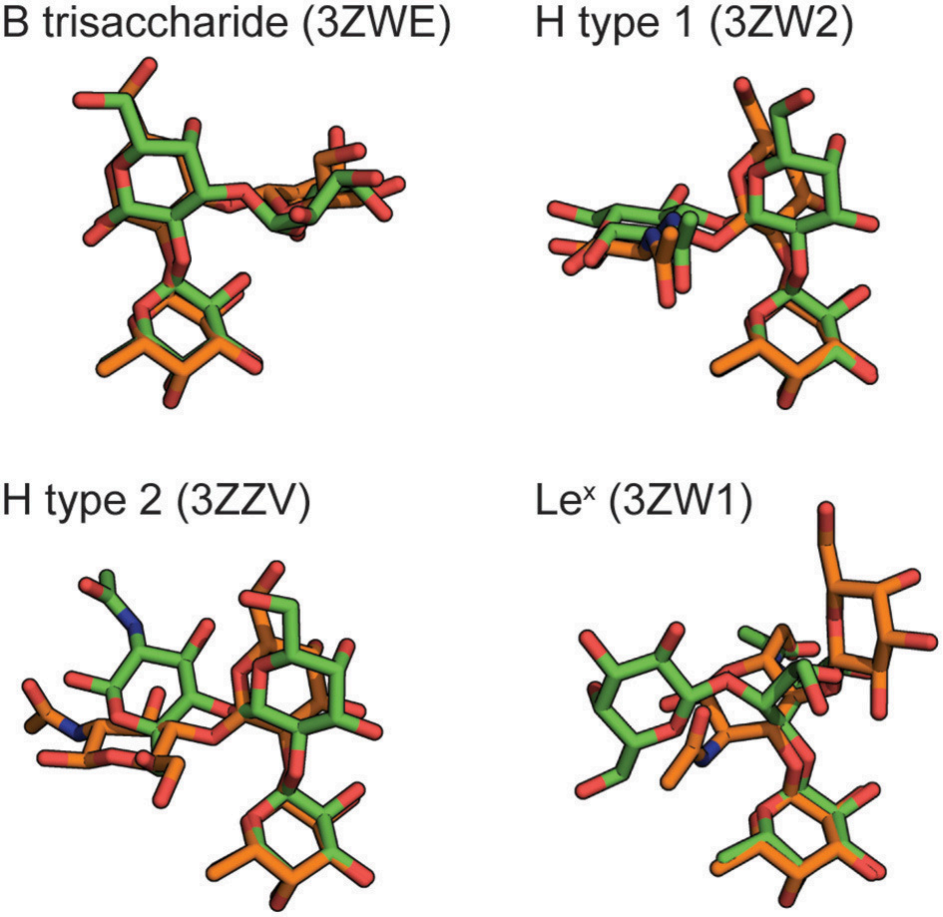
Blood group carbohydrates docked into the BambL binding site of PDB ID: 3ZZV (orange), compared to their respective experimentally determined poses (green). The PDB IDs for experimental poses are indicated. For clarity, all carbohydrates are shown as the non-reducing-end trisaccharide without hydrogen atoms.

To decide which of the crystallographic BambL receptor structures to use in this study, we compared complex structures predicted by re-docking with respective crystallographic complexes. The results of these cognate and cross-docking experiments are shown in Table 1, Figure 2. The Le^x^ tetrasaccharide was poorly docked (RMSD > 2 Å) into all BambL structures. However, all four lectin structures afforded approximately equal performance when used as a receptor for the other three carbohydrate ligands: overall RMSD values of 1.09–2.62 and 0.14–0.56 Å for the buried fucose (Fucα1-2Gal) were observed. The crystallographic BambL structure from the PDB ID: 3ZZV complex was used as the receptor structure for site mapping and MD with all carbohydrates shown in Figure 1.

**Table 1:** Top scoring docked pose characterization for BambL-blood group saccharide complexes.

	**RMSD of top docked pose to crystal structure** **(Å)^a^**			
**Ligand**	**3ZWE (1.75 Å)**	**3ZW2 (1.60 Å)**	**3ZZV (1.68 Å)**	**3ZW1 (1.60 Å)**
B^b^	**1.68 (0.42)**^c^	2.04 (0.25)	2.13 (0.36)	2.62 (0.28)
H1	1.80 (0.47)	**1.09 (0.29)**^c^	1.47 (0.14)	2.02 (0.27)
H2^b^	1.90 (0.39)	2.42 (0.39)	**1.56 (0.25)**^c^	1.52 (0.56)
Le^x^	9.47 (7.95)	4.61 (0.58)	6.94 (10.31)	**7.01 (0.32)**^c^

a *The experimental resolution of each crystallographic BambL complex is shown in brackets beneath the PDB ID. RMSD values compare the ligand portion common between the docked and crystallographic ligand; RMSD values in brackets compare the fucose portion of the docked ligand to the fucose portion of the crystallographic ligand*.

b *Cross-docking performed using the ligands used in site mapping and molecular dynamics (Figure 1). Cognate docking performed using the ligand length present in the crystallographic complex*.

c *Values shown in bold indicate cognate docking experiments*.

**Figure 3:**
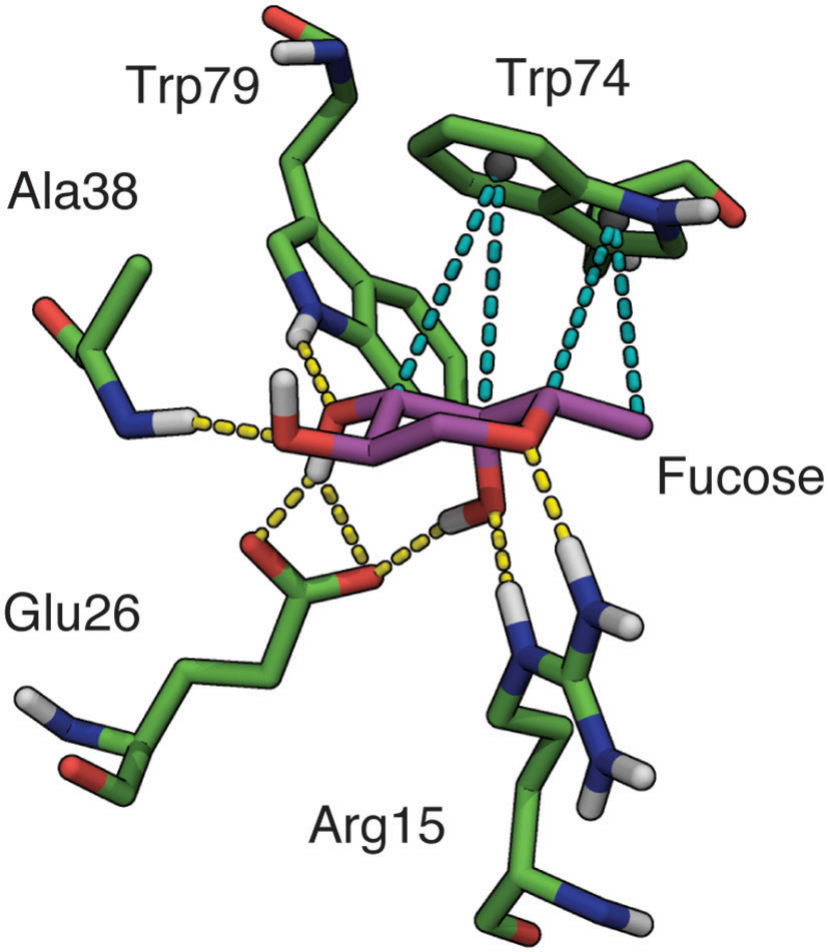
Binding site interactions involving fucose in BambL-blood group saccharide docked poses. Hydrogen bonds shown as yellow dashes, hydrophobic interactions shown as teal dashes. Non-polar hydrogens omitted for clarity.

In a second step, all blood group saccharides were docked in BambL (PDB ID: 3ZZV) and the top docked poses were analyzed for structural features relevant to recognition (Table 2). In all cases except Le^x^, the majority of binding interactions were made via a single buried fucose residue (Figure 3). The difucosylated Le^b^ and Le^y^ possess two fucose residues (Fucα1-2Gal and Fucα1-4GlcNAc in Le^b^ or Fucα1-3GlcNAc in Le^y^) and therefore may occupy the fucose-binding pocket in two ways. Of the docked Le^b^ poses produced here, only the Fucα1-2Gal residue was predicted in the binding pocket. As for the docked Le^y^ poses, all of the top 20 ranked poses positioned the Fucα1-2Gal residue in the pocket, with the exception of poses at rank 5 and 6 that predicted the Fucα1-3GlcNAc residue in the fucose binding pocket.

**Table 2:** Top scoring docked pose characterization for BambL-blood group saccharide complexes.

**Ligand**	**Fucose RMSD (Å)^a^**	**Glycosidic dihedral angles^b^**						**Hydrogen bonds^c^**	**Docking score (kcal/mol)**
		**φ**	**ψ**	**φ**	**ψ**	**φ**	**ψ**		
A tri	0.53	Fucα1-2Gal		GalNAcα1-3Gal				GalNAc2-H6O…Asp30-OD1	−5.848
		−106.8	55.9	81.3	76.8			GalNAc2-O4…Trp8-HE1	
B tri	0.24	Fucα1-2Gal		Galα1-3Gal				Gla3-H6O…Asp30-OD1	−5.890
		−107.9	63.9	49.5	45.8			Gla3-O3…Trp74-HE1	
H1	0.43	Fucα1-2Gal		Galβ1-3GlcNAc		GlcNAcβ1-3Gal		GlcNAc2-H4O…Asp30-OD2	−6.879
		−116.2	−129.6	−42.2	146.6	−106.1	159.2	GlcNAc2-O2N…Trp74-HE1	
H2	0.38	Fucα1-2Gal		Galβ1-4GlcNAc		GlcNAcβ1-3Gal		Gal1-H3O…Tyr35-O	−6.597
		−106.0	−88.0	−42.2	−91.8	−81.8	101.0	Gal1-H6O…Asp77-OD2	
								GlcNAc2-H6O…Gly76-O	
Le^a^	0.30	Fucα1-4GlcNAc		Galβ1-3GlcNAc		GlcNAcβ1-3Gal		Gal4-H2O…Asp30-OD2	−5.706
		−138.3	−147.3	−64.8	138.7	−68.1	96.6		
Le^x^	10.22	Fucα1-3GlcNAc		Galβ1-4GlcNAc		GlcNAcβ1-3Gal		Gal1-O5…Ala38-H	−5.786
		−76.4	151.5	−72.6	−112.7	−66.5	89.6	Gal1-O6…Trp79-HE1	
								Gal1-H6O…Glu26-OE1	
								Gal1-O4…Arg15-HH21	
								Gal1-H4O…Tyr35-OH	
								Fuc4-H2O…Asp30-OD2	
								Fuc4-H3O…Asp30-O	
								Gal3-H2O…Ser55-O	
								Gal3-O6…Thr11-HG1	
								Gal3-H6O…Ser13-OG	
Le^b^	0.38	Fucα1-2Gal		Fucα1-4GlcNAc		Galβ1-3GlcNAc		Fuc2-H2O…Asp30-OD1	−7.065
		−83.6	−98.8	−70.3	96.5	−39.5	166.6	Fuc2-O3…Trp8-HE1	
								GlcNAc1-O6…Trp74-HE1	
								GlcNAc1-H6O…Val57-O	
								GlcNAc1-HO1…Gly76-O	
Le^y^	0.33	Fucα1-2Gal		Fucα1-3GlcNAc		Galβ1-4GlcNAc		Fuc2-H2O…Asp30-OD2	−7.007
		−105.3	−138.3	−83.8	−51.3	−56.0	−103.7	GlcNAc1-H2N…Asp30-OD1	
								GlcNAc1-O1…Trp8-HE1	
								GlcNAc1-O6…Trp74-HE1	

a *Calculated for buried fucose residue heavy atoms between crystallographic saccharide (PDB ID: 3ZZV) and docked ligand*.

b *Dihedral angles defined as: φ, O_5_-C_1_-O_1_-C_x_; ψ, C_1_-O_1_-C_x_-C_x+1_*.

c *Excluding hydrogen bonds involving the buried fucose residue*.

As expected, recognition of the buried fucose (Fucα1-2Gal) was governed by a conserved hydrogen-bonding network and a single hydrophobic stacking interaction (Supplementary Table 1). Rather than interacting via a buried fucose, the Le^x^ top docked pose was placed “back-to-front” with the reducing end galactose in the fucose-binding pocket, and the fucose directed away from the protein.

Apart from interactions with the buried fucose residue, additional hydrogen bonds are made between non-fucose residues and amino acids in the four β-turn loops surrounding the fucose-binding pocket (Table 2). The most frequently participating residue, Asp30, interacts with non-fucose portions of multiple saccharides (B, H1, Le^a^, Le^b^, and Le^y^). The imidazole side-chain of Trp74 (which stacks against the buried fucose) also donates a hydrogen bond to non-fucose residues in several cases. In each case, the hydrogen bond is accepted by atoms in a similar location: two residues away from the buried fucose, at the GlcNAc 6-position (Le^b^, Le^y^), Gal/GalNAc 3-position (A, B), or GlcNAc 2-position (H1). The presence of hydrogen bonds between non-fucose portions and loop residues suggests that BambL recognition may not rely solely on interactions with a single buried fucose.

Glycosidic dihedral angles in top docked poses lie close to global or secondary minima in previously calculated (Imberty et al., 1995) energy maps (see Supplementary Figures 2, 3). An exception is the Fucα1-2Gal linkage, which is positioned in between minima in the H type 1, H type 2, Le^b^ and Le^y^ top poses. In the A and B trisaccharide complexes, the Fucα1-2Gal linkage adopted the lowest energy conformation. These results agree with earlier BambL-blood group docking by Topin et al. (2013) in which top docked pose glycosidic linkages also occupied a range of energetic minima.

### Site mapping of BambL-blood group complexes

**Figure 4:**
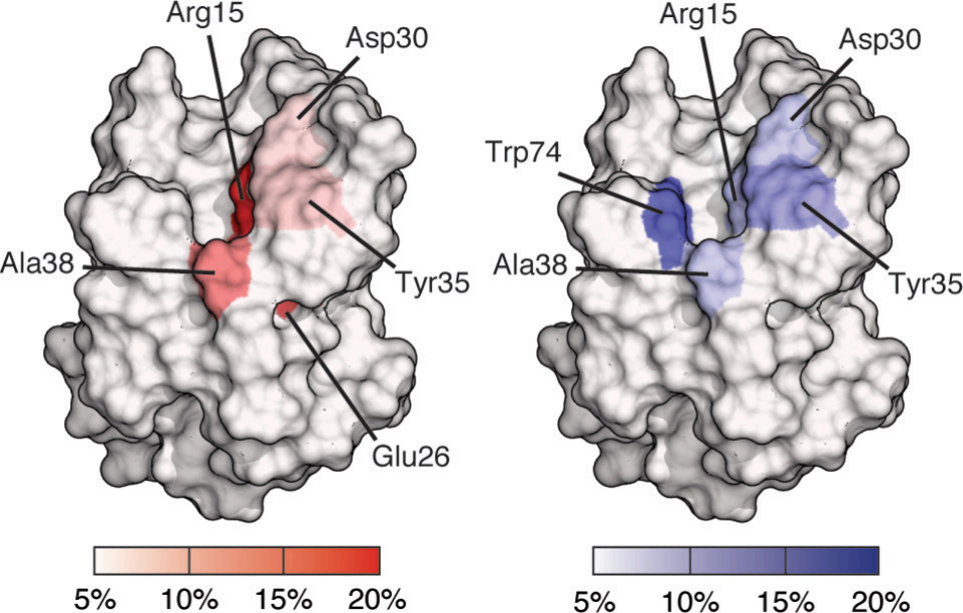
Site maps of a BambL subunit showing binding site residues involved in docked pose interactions. Residues involved in 5% or fewer interactions are colored white; residues involved in 20% or greater interactions are colored red (for hydrogen bonding) or blue (for van der Waals). Residues with intermediate involvement are shaded according to the color scale.

Site mapping reveals binding site residues that are frequently involved in interactions throughout an ensemble of docked poses. Site maps for BambL-blood group complexes are shown in Figure 4. These maps are based on docking results for all carbohydrates shown in Figure 1. The BambL site maps agree with crystallographic complexes, identifying multiple residues in the fucose binding pocket known to interact with fucose in crystallographic structures (PDB IDs: 3ZW2, 3ZZV, 3ZWE, and 3ZW1; Audfray et al., 2012; Topin et al., 2013). Across the docked pose ensemble, hydrogen bonds were frequently formed to Arg15 (27.9%), Ala38 (11.6%), and Glu26 (13.7%), all located within the fucose-binding pocket. Surprisingly, Trp79 (4.9%), also in the crystallographic fucose pocket, was not often involved throughout the docked pose ensemble. van der Waals (vdW) interactions were frequently made with Trp74 (14.6%) in the fucose pocket, in close agreement with crystallographic bound complexes. Site maps also revealed new interactions not seen in crystal structures, identifying hydrogen bonding to Asp30 (7.1%) and vdW interaction with Tyr35 (11.1%) as regularly occurring across all docked poses.

### Molecular dynamics simulations of BambL-blood group complexes

To investigate the dynamic behavior of BambL-blood group complexes, the lowest-energy poses generated by docking were simulated in explicit solvent. For difucosylated Le^b^ and Le^y^, the lowest-energy poses with the Fucα1-2Gal residue in the fucose-binding pocket were used. The poorly docked Le^x^ complex was also simulated, but quickly dissociated from the protein or was unstable in the binding site (see Supplementary Figure 4). To probe the dynamic behavior of the Le^x^ binding interactions, the crystallographic complex was used instead (PDB ID: 3ZW1).

**Figure 5:**
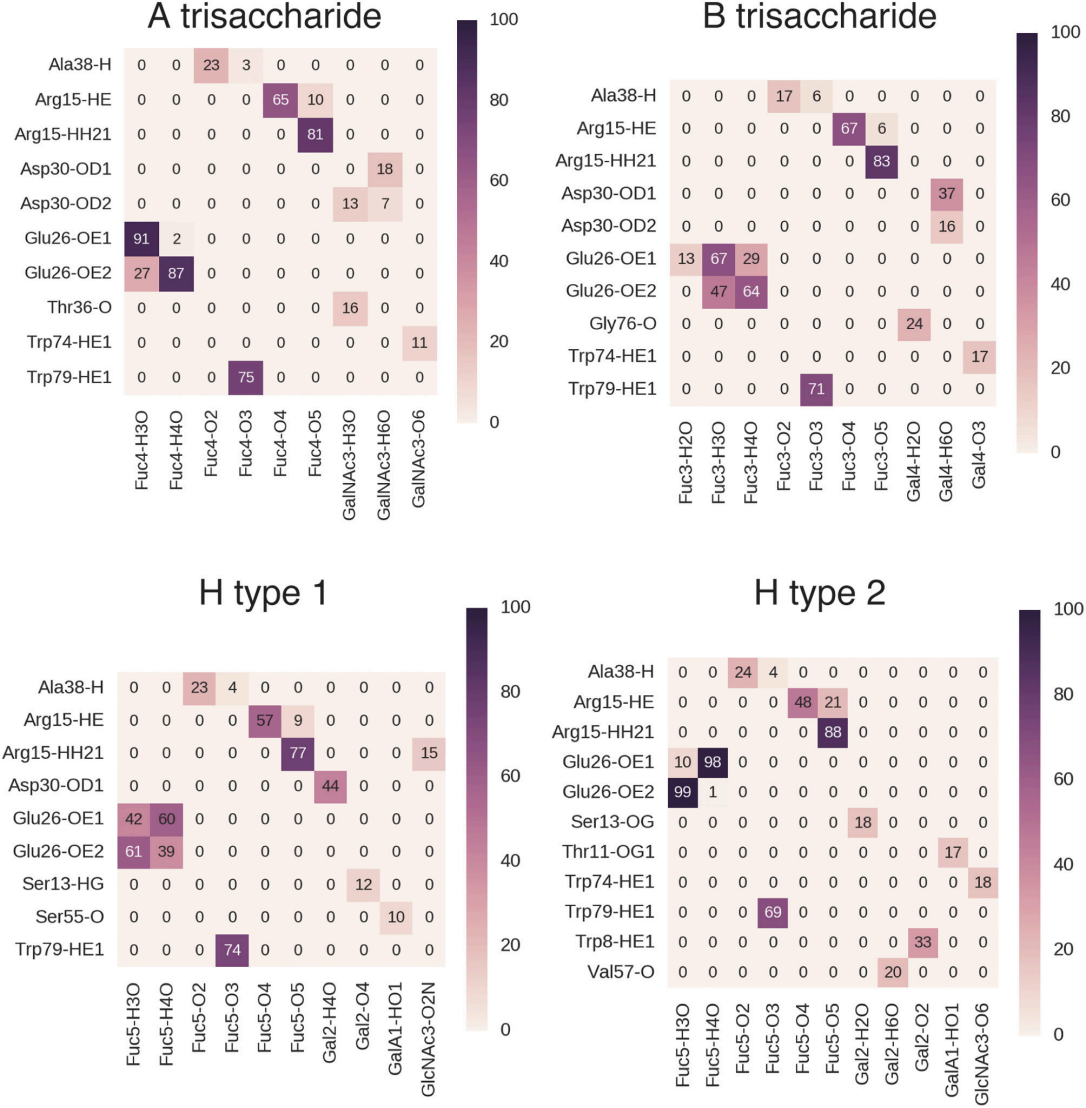
Hydrogen bond interactions in BambL-saccharide complexes during MD simulations shown for A trisaccharide, B trisaccharide, H type 1 and H type 2 blood groups. Atoms are named to the conventions of the PDB exchange dictionary. Grid cells are colored and labeled by average occupancy from three replicate simulations. Occupancy values were calculated by dividing the number of frames in which the hydrogen bond exists by the total number of simulation frames.

**Figure 6:**
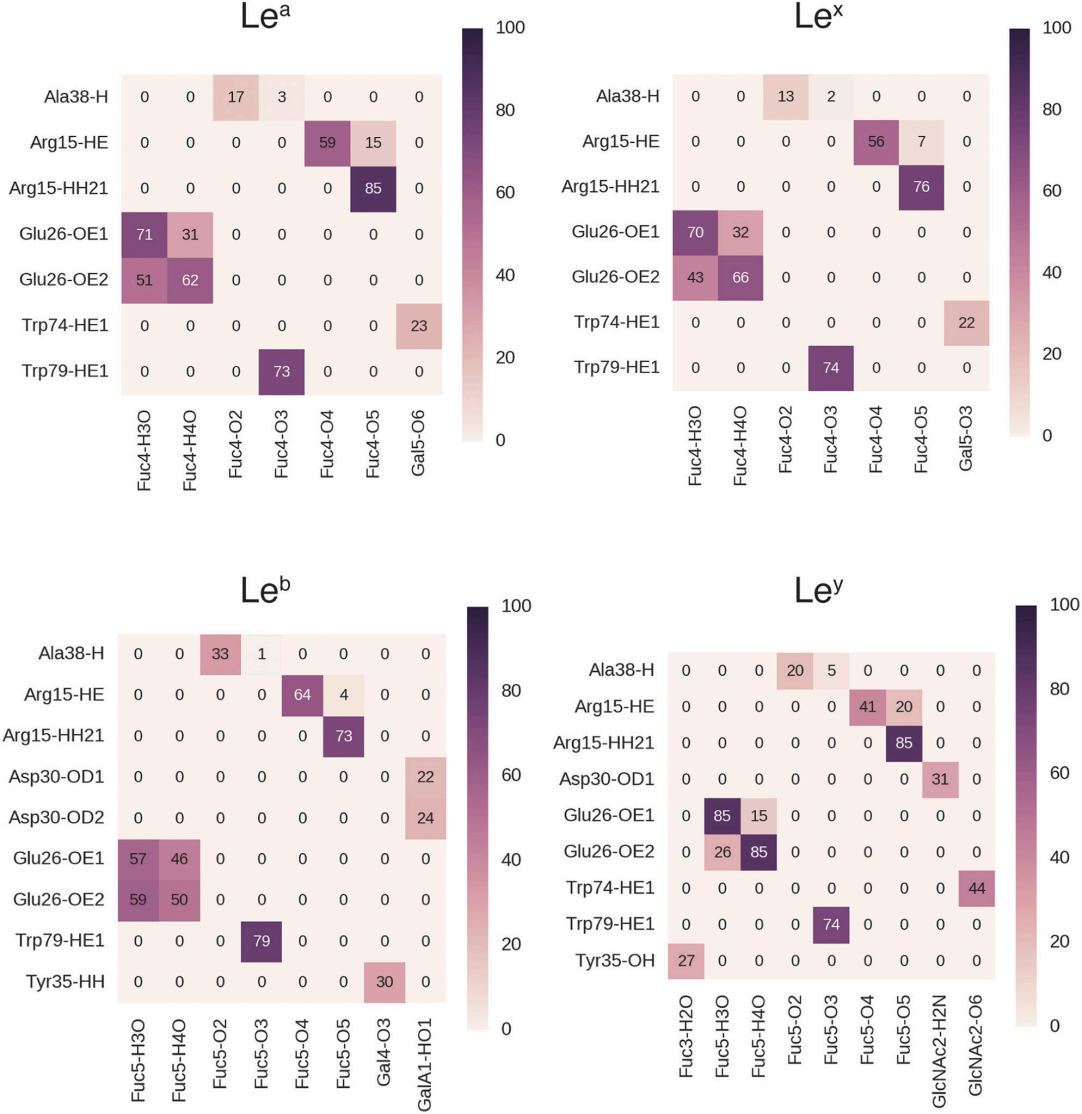
Hydrogen bond interactions in BambL-saccharide complexes during MD simulations shown for Lewis group saccharides. Atoms are named to the conventions of the PDB exchange dictionary. Grid cells are colored and labeled by average occupancy from three replicate simulations. Occupancy values were calculated by dividing the number of frames in which the hydrogen bond exists by the total number of simulation frames.

During MD simulations, all fucose-anchored blood group saccharides (A, B, H type 1, H type 2, Le^a^, Le^b^, Le^y^) remained bound to BambL without dissociation for the entire duration (400 ns). Structural fluctuations in ligand RMSD were below 2 Å in all bound complexes, reflecting relatively small changes in ligand positions and geometries during the MD simulations (see Supplementary Figure 5). Carbohydrate ring conformations were found to generally adopt one of the two chair conformations (^1^C_4_ or ^4^C_1_), while the GlcNAc rings in the H type 2, Le^a^, and Le^x^ exhibited some variation (see Supplementary Figure 6). A similar hydrogen-bonding pattern was observed across all blood group simulations (Figures 5, 6), featuring interactions between the buried fucose residue and the fucose-binding pocket: Glu26 acidic group to O3 and O4 hydroxyl protons, Arg15 guanidinium to O4 and O5 oxygen atoms, and Trp79 indole to O3 oxygen atom. These hydrogen bonds were highly occupied (between 60 and 90% of simulation frames), with the exception of the Glu26 hydrogen bonds in the Le^b^ complex (50–60%). The high occupancy of these hydrogen bonds indicates the dominant role played by fucose in BambL-carbohydrate binding.

In addition to the above interactions, a low-occupancy (up to 30% of simulation frames) hydrogen bond was observed between the Ala38 backbone amide proton and the buried fucose 2-position hydroxyl oxygen atom. In contrast to the highly occupied hydrogen bonds, this interaction engages a backbone proton rather than a side-chain; combined with the low occupancy, this suggests a less significant contribution by this hydrogen bond to carbohydrate binding. Alongside hydrogen-bonding interactions, stacking of the fucose C3-C4-C5-C6 hydrophobic face against the Trp74 indole ring was consistently maintained during simulation (see Supplementary Figure 7).

Hydrogen bonds to non-fucose portions of the carbohydrate ligands were formed at low to moderate occupancies (20–50%) with fucose-binding residue Trp74 (Le^y^: 44%, Le^a^: 23%, Le^x^: 22%) and surface residue Asp30 (B: 37%, H type 1: 44%, Le^b^: 24%, Le^y^: 31%).

**Figure 7:**
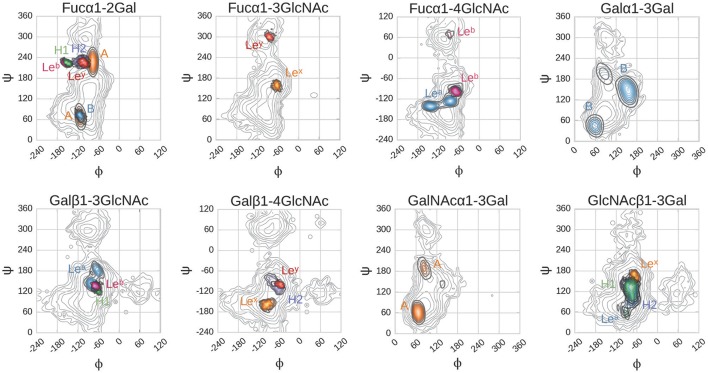
Glycosidic dihedral angles in BambL-blood group saccharide complexes during MD simulations. Dihedrals are defined as: φ, O_5_-C_1_-O_1_-C_*x*_; ψ, C_1_-O_1_-C_*x*_-C_*x*+1_. Contour plots color coding: gray, calculated energy landscapes of constituting linkages; brown, A; light blue B; green H1; dark blue, H2; cyan Le^a^; pink, Le^b^; orange Le^x^; red Le^y^. Contour plot lines mark intervals of 1 kcal/mol.

Glycosidic linkage conformations explored during MD simulations occupy global, and occasionally secondary, minima (Figure 7). As observed in docking, the Fucα1-2Gal linkage is again an exception, adopting a position intermediate between the two minima for the entire duration of simulation in the H type 1, H type 2, Le^b^ and Le^y^ complexes. In the H type 1 and Le^b^ complexes, this linkage explores a narrower range of higher-energy conformations compared to H type 2 and Le^y^. It is possible that this difference between the calculated energetic minima and the conformations observed in simulation is due to the presence of the protein. Force field-based energy contours describe the energetic behavior of each linkage as an unbound disaccharide in vacuum (Imberty et al., 1995), while simulation of the bound complex introduces protein, water, and other saccharide units within the tri- or tetrasaccharide, all of which influence conformational behavior. A recent example of the influence of protein binding on carbohydrate conformation is the Le^x^ saccharide, which occupies well-characterized “closed” conformations in solution and “open” conformations when bound to the RSL lectin (Topin et al., 2016; defined by the relative positions of the fucose and galactose rings). In the present study, the Le^x^ saccharide maintained an open conformation during MD simulation, corresponding to shapes “Open V” and “Open II” in the scheme defined by Topin et al. (2016) consistent with its continuous occupation of the binding site during simulation (see Supplementary Figure 8).

In the A and B trisaccharide simulations, the *N*-acetylgalactosamine and non-reducing end galactose move more freely than the saccharide occupying the same position in the other ligands. The Fucα1-2Gal glycosidic linkage in these two saccharides occupies two conformations, defined by variation in the ψ-angle between −60° and +100°. The A trisaccharide explores both, while the B trisaccharide only occupies the former conformation (Figure 7).

### Docking and MD simulations of complexes with blood-group-related carbohydrates

Interactions between BambL and blood group/tissue carbohydrates was mediated mainly via the single buried fucose, with occasional hydrogen bonds formed between non-fucose atoms and residues on loops surrounding the binding pocket. Identifying these non-fucose binding interactions may provide opportunities to improve inhibitor affinity for BambL beyond the current fucose-based inhibitors.

The potential for non-fucose binding interactions to form in BambL-saccharide complexes was explored by simulating complexes of 36 blood-group-related carbohydrates to the protein (i.e., a focused carbohydrate library). The related carbohydrates ranged in size from di- to heptasaccharides and were composed of fragments of blood group and tissue determinant carbohydrates and elongated versions of blood group carbohydrates bearing additional saccharides (for structures of all library members, see Supplementary Figure 1). Most of these structures contain fucose moieties and were expected to interact with BambL via the fucose-dominated mode observed in crystallographic structures. To explore how non-fucose residues (such as galactose and *N*-acetylgalactosamine) might occupy the fucose-binding site, a selection of di- and trisaccharides lacking fucose were also evaluated. Complexes with BambL were assembled by docking and simulated in explicit solvent for 400 ns.

**Figure 8:**
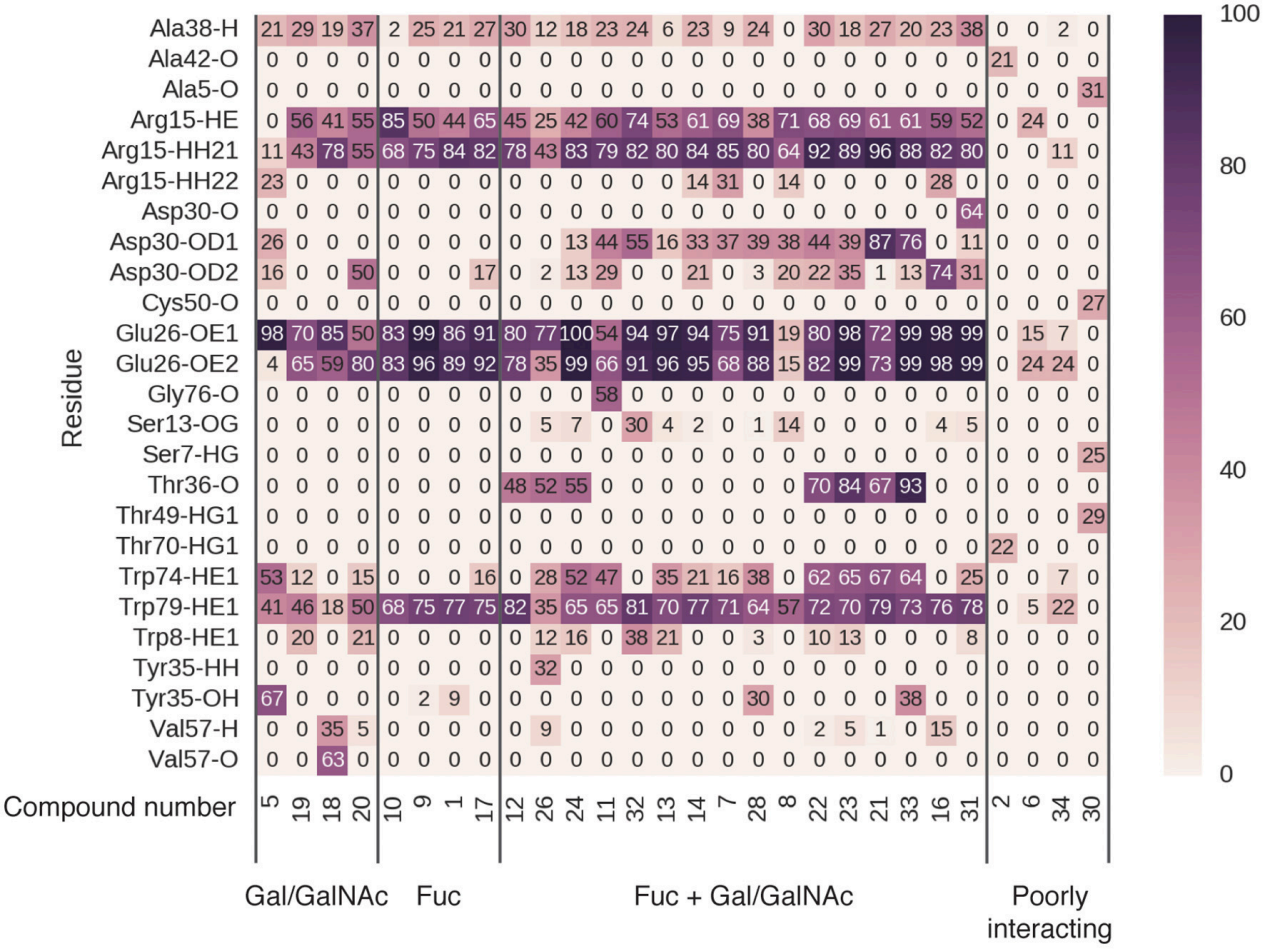
Hydrogen bonding occupancy of blood-group-related saccharides during MD simulations. Saccharide names indicate the ligand moieties interacting with BambL during simulation.

Of the 36 complexes simulated, 28 remained stably engaged without dissociation of the ligand into bulk solvent. Multiple binding modes were observed among the stable complexes, exhibiting different hydrogen-bonding patterns (Figure 8). In some complexes (**2, 6, 34, 30**), very few hydrogen bonds were formed and were observed for only up to 30% of MD runs. These binding modes, while stable, did not feature significant hydrogen-bonding interactions with BambL.

In four cases (**5, 19, 18, 20**), the ligand was found to interact with the fucose-binding pocket via a non-fucose saccharide (galactose or *N*-acetylgalactosamine). While these non-fucose binding modes do include hydrogen bonds to the three fucose pocket residues (Arg15, Glu26, and Trp79), these interactions are not as highly occupied as those made by fucose-containing saccharides (**10, 9, 1, 17**). In non-fucose binding modes, hydrogen-bond occupancies over 70% were observed for only one or two interactions per ligand; for fucose-mediated binding, all three pocket residues are engaged more than 70% of the time.

The remaining 20 carbohydrates bound in a fucose-dominated manner, forming hydrogen bonds at over 70% occupancy between a fucose and all three residues of the fucose-binding pocket. In most cases, additional hydrogen bonds were formed with loop residues outside the fucose-binding pocket, with occupancies ranging from 10 to 90%. The highly stable (>70% occupancy) non-fucose hydrogen bonds involved residues Asp30 and Thr36, located on loop 4. The acidic sidechain of Asp30 projects toward the fucose-binding pocket, accepting hydrogen bonds from saccharides not directly bonded to the buried fucose. Thr36 is located further away from the fucose-binding pocket, and accepts hydrogen bonds via the backbone carbonyl oxygen atom. A less-occupied hydrogen bond (up to 67%) is formed to the indole nitrogen of Trp74, concurrent with hydrophobic stacking against a buried fucose. Finally, Tyr35 donates a hydrogen bond via the phenolic hydroxyl to compound **28** and **33** (and additionally to the non-fucose compound **5**). The fucose-dominated binding modes featuring highest occupancy of non-fucose hydrogen bonds involved carbohydrates **21** and **33**, illustrated in Supplementary Figure 9.

**Figure 9:**
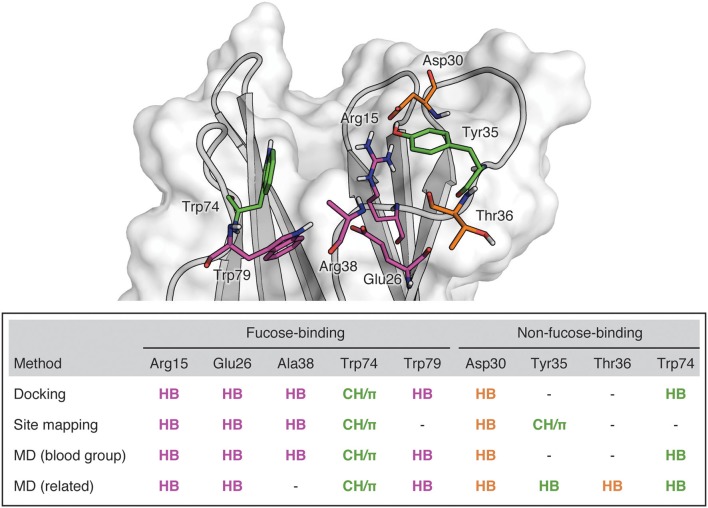
BambL binding site showing residues implicated in saccharide binding. Purple: Residues which form hydrogen bonds with the buried fucose saccharide. Orange: Residues which form hydrogen bonds with non-fucose saccharides. Green: Residues which participate in both hydrophobic and hydrogen bonding interactions.

Combining all the BambL residues involved in hydrogen bonds to fucose and non-fucose saccharides presents a perspective of the target site that incorporates a wider view of BambL-saccharide recognition, considering multiple interaction points across the protein surface (Figure 9). This view of the BambL binding site presents opportunities for future inhibitor design to consider regions outside the fucose-binding pocket.

## Discussion

We have investigated the molecular aspects of carbohydrate recognition of the *B. ambifaria* lectin by computational methods: docking, site mapping, and MD. Molecular docking has been shown to be extremely useful for structural predictions, if not affinity calculations (Yuriev et al., 2015). However, docking carbohydrate ligands presents a number of challenges stemming from their extreme flexibility, a large number of hydroxyl groups, leading to the formation of (often) extensive hydrogen-bonding networks, and the formation of crucial CH/π stacking interactions between the C-H bonds of the carbohydrates (on their hydrophobic faces) and aromatic side chains of the protein (Agostino et al., 2009a, 2012a). Also, carbohydrate ligands are modular, and different residues (e.g., galactose vs. glucose) are able to establish highly similar interactions with the binding site. We have previously validated Glide and tested a range of other docking programs for structural prediction of carbohydrate complexes with antibodies (Agostino et al., 2009a, 2012b) and lectins (Agostino et al., 2011). We have demonstrated that, as the result of all the above-mentioned challenges, docking programs and scoring functions are not always able to predict the native binding pose faithfully as the top docked pose. To overcome this shortcoming and to harness the recognition information embedded in the docking output, we have developed a site mapping methodology that takes into account an ensemble of docked poses and identifies binding site residues critically involved in recognition of a ligand or ligand family (Yuriev et al., 2001, 2002; Agostino et al., 2013; Dingjan et al., 2015a).

In this study, docking with Glide produced reasonable top poses for a range of BambL complexes with blood group carbohydrates (Table 2). Using the BambL structure from PDB ID: 3ZZV gave accurate complex prediction for the B, H type 1 and H type 2 saccharides and accurate fucose placement for the A, Le^a^, Le^b^, and Le^y^ determinants. All these complexes featured a buried fucose residue (Fucα1-2Gal), providing the majority of hydrogen-bonding interactions, and conformational ranges reflective of predicted energetic minima (Imberty et al., 1995) and relevant experimental structures (Yuriev et al., 2005; Dingjan et al., 2015b). Notably, the distances between fucose carbon atoms and the geometric centers of the imidazole phenyl and pyrrole component ring systems of Trp74 (Supplementary Material, Table S1) are similar to reported geometries for fucose CH/π dispersion interactions of a closely related lectin, RSL (Wimmerova et al., 2012). As in the RSL-fucose complex, the C6 atom interacts with the pyrrole part of the imidazole ring (distance of 3.76 ± 0.3 Å), while C3 is further than 4 Å away. Unlike the RSL complex, C5 also interacts with the pyrrole ring (distance of 3.83 ± 0.1 Å), rather than the phenyl ring, which is further than 4 Å from the entire non-polar plane.

Detailed elaboration of structural aspects of molecular recognition requires expanding the single snapshot view afforded by crystal structures or top docked poses. To that effect, we have undertaken site mapping and MD investigations in order to identify BambL residues critical for recognition of blood group carbohydrates. The advantage of site mapping lies in its ability to consider alternative binding modes while MD also explicitly accounts for the role of water, mediating interactions of BambL to carbohydrates.

We have identified the atomic scale binding interactions that facilitate recognition of fucosylated human blood group saccharides by BambL. A network of hydrogen bonds combined with a single hydrophobic stacking interaction between the buried fucose and amino acids in the fucose-binding pocket account for the majority of binding interactions (Figure 3). These structural features of the fucose-driven recognition closely agree with experimental characterization of BambL-carbohydrate binding profile by glycan array, which has demonstrated a preference for short, fucose-bearing saccharides, with the fucose monosaccharide among the most highly ranked binders (Audfray et al., 2012). However, this fucose-driven recognition motif does not explain the specificity profile of BambL compared to other related fucose-binding lectins. Namely, the interactions between BambL and fucosylated saccharides are highly similar to those found in complexes featuring other six-bladed β-propeller fucose-binding lectins: found in fungi [*Aleuria aurantia* lectin, AAL (Fujihashi et al., 2003; Wimmerova et al., 2003); *Aspergillus fumigatus* lectin, AFL (Houser et al., 2013); *Aspergillus oryzae* lectin, AOL (Makyio et al., 2016)] and bacteria [*Ralstonia solanacearum* lectin, RSL (Kostlánová et al., 2005)]. Members of this lectin family bind fucose via the same interactions: hydrogen bonds between O2 and a backbone amide proton, O3 and indole nitrogen, O3 and O4 to a shared carboxylate moiety, and O4 and O5 to a shared guanidinium moiety. In a previous docking study of RSL-fucose recognition by Mishra et al. (2012) the same suite of interactions was reported.

Despite the common binding mode, these lectins prefer different blood group determinants: AAL exhibits broad specificity, while AFL prefers Le^y^, and RSL prefers saccharides featuring Fucα1-2 and Fucα1-6 moieties (blood group A, B, and H and core of N-glycans). Varied blood group specificity has been proposed to arise from steric hindrance around the fucose-binding pocket, preventing strong binding to most branched carbohydrate structures (Fujihashi et al., 2003). Glycan array screening shows generally decreased binding to branched carbohydrates compared to mono- and disaccharides for these lectins, emphasizing the importance of steric effects (Houser et al., 2013). Additionally, the non-selective AAL lacks steric hindrance around the fucose-binding pocket: in a bound complex featuring the disaccharide Fucα1-6GlcNAcβ1-OMe, transferred NOE experiments confirmed conformational flexibility around the glycosidic linkage (Weimar and Peters, 1994). However, steric hindrance alone does not fully explain blood group selectivity in this lectin family. AFL binds the difucosylated Le^y^ more strongly than the corresponding monofucosylated saccharide, H-type 2, despite similar steric complementarity to the binding site (Houser et al., 2013). We suggest that stabilizing interactions outside the fucose-binding pocket (as observed in simulations of BambL complexed with blood-group-related saccharides) play a role in saccharide binding in the 6-bladed β-propeller lectin family more generally.

Interactions with non-fucose residues are not as highly occupied as interactions with the fucose. However, they contribute to a wider view of BambL-carbohydrate recognition, considering multiple interaction points across the protein surface. They include hydrogen bonding to Asp30, Tyr35, Thr36, and Trp74 and hydrophobic contacts with Tyr35 (Figure 9). These contacts outside the fucose-binding pocket could be employed in future inhibitor design for BambL to address issues of opportunistic infections.

## Conclusion

In summary, the present work details the recognition of fucosylated human blood group determinants by BambL, quantifies the occupancy of hydrogen bonding interactions, and identifies opportunities for targeting residues outside the fucose-binding pocket. Recognition mainly involves the fucose monosaccharide through a network of highly occupied hydrogen-bonding interactions to Arg15, Glu26, and Trp79, and a lower occupancy interaction with Ala38. An additional stacking interaction between the fucose hydrophobic face and Trp74 is also highly occupied in MD simulations. Hydrogen bonds to non-fucose saccharides were formed in complexes with Le^y^, Le^b^, Le^a^, H1, H2, and B trisaccharide and in multiple complexes involving blood-group-related saccharides. The most occupied interactions involved Asp30, Thr36, Trp74, and to a lesser degree Tyr35. Carbohydrate recognition by BambL is therefore proposed to be driven by interactions in the fucose-binding site and further stabilized by satellite interactions between non-fucose saccharides and surface residues outside the fucose-binding pocket. The analysis of carbohydrate recognition by BambL presented in this study lays the foundation for the development of fucomimetic molecules able to bind to BambL. Such molecules have potential as anti-adhesives for the treatment of *B. ambifaria* infection in cystic fibrosis patients.

## Author contributions

Each author has contributed significantly to the submitted work. TD and EY conceived and designed the experiments. TD performed the experiments. TD, EY, and PR analyzed the data. TD, AI, SP, EY, and PR wrote the paper. All authors read and approved the final manuscript.

### Conflict of interest statement

The authors declare that the research was conducted in the absence of any commercial or financial relationships that could be construed as a potential conflict of interest.

## Abbreviations

BambL: *Burkholderia ambifaria* lectin
H1: H type 1
H2: H type 2
Le^a^: Lewis a
Le^b^: Lewis b
Le^x^: Lewis x
Le^y^: Lewis y
MD: Molecular Dynamics
PDB: Protein Data Bank
vdW: van der Waals
RMSD: root mean square deviation
