# Supplementary material for: Molecular Simulations of Carbohydrates with a Fucose-Binding Burkholderia ambifaria Lectin Suggest Modulation by Surface Residues Outside the Fucose-Binding Pocket
Source: Front Pharmacol. 2017 Jun 21;8:393. doi: 10.3389/fphar.2017.00393 (PMC5478714; doi:10.3389/fphar.2017.00393)
Supplement: Supplementary file 1 [file DataSheet1.PDF]

Supplementary Material

Molecular Simulations of Carbohydrates with a Fucose-Binding *Burkholderia ambifaria* Lectin Suggest Modulation by Surface Residues Outside the Fucose-Binding Pocket

Tamir Dingjan[1], Anne Imberty[2], Serge Pérez[3], Elizabeth Yuriev[1]*, Paul A. Ramsland[4,5,6,7]*

[1]Medicinal Chemistry, Monash Institute of Pharmaceutical Sciences, Monash University, Melbourne, VIC, Australia.

[2]Centre de Recherches sur les Macromolécules Végétales, CNRS UPR5301, Université Grenoble Alpes, Grenoble, France.

[3]Département de Pharmacochimie Moléculaire, CNRS UMR5063, Université Grenoble Alpes, Grenoble, France.

[4]School of Science, RMIT University, Melbourne, VIC, Australia.

[5]Department of Surgery Austin Health, University of Melbourne, Melbourne, VIC, Australia.

[6]Department of Immunology, Central Clinical School, Monash University, Melbourne, VIC, Australia.

[7]Burnet Institute, Melbourne, VIC Australia.

* Correspondence:

Dr Paul A Ramsland: paul.ramsland@rmit.edu.au

Dr Elizabeth Yuriev: elizabeth.yuriev@monash.edu

1 Supplementary Figures and Tables

1.1 Supplementary Figures

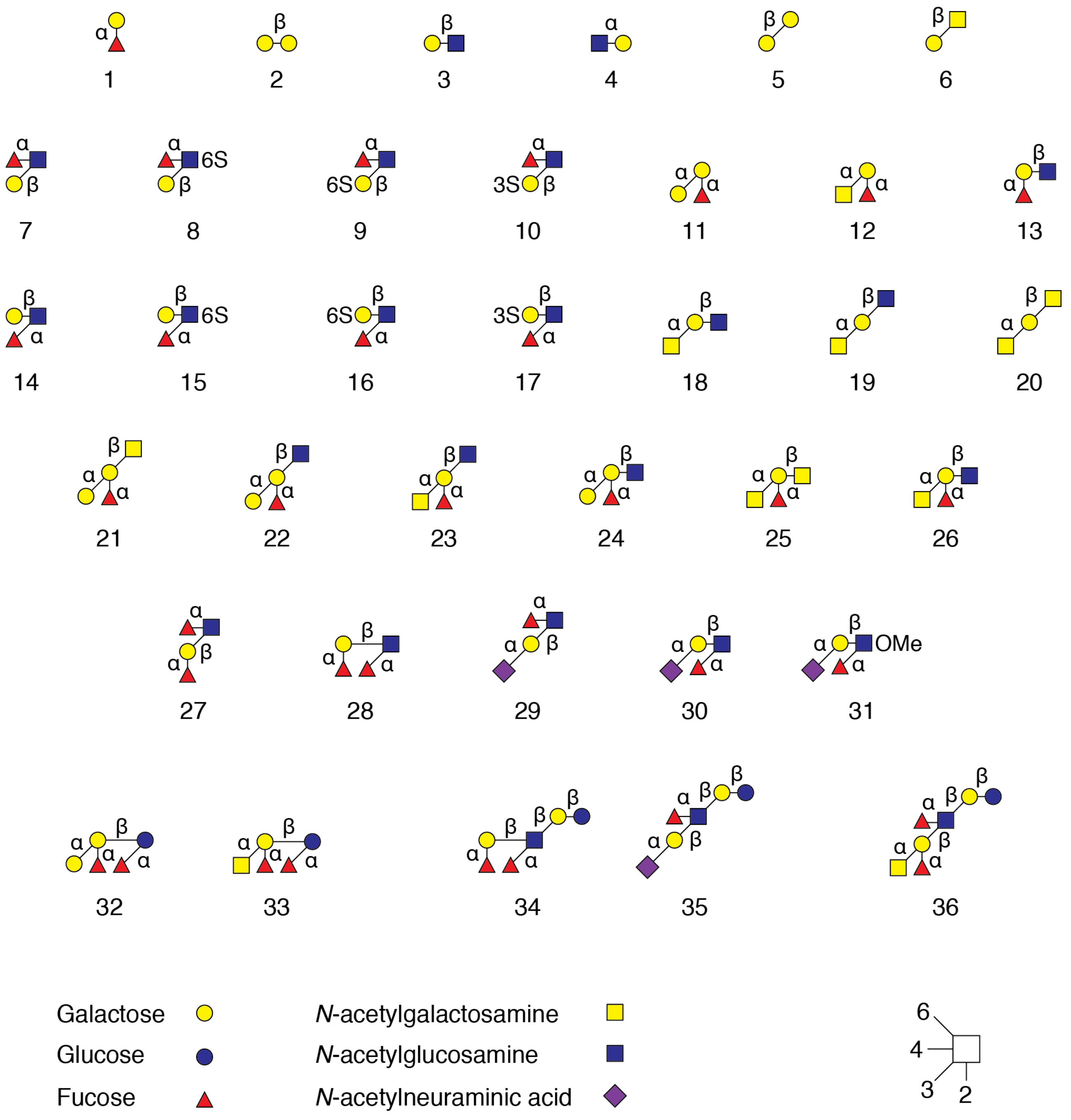

Supplementary Figure 1. Blood-group-related carbohydrates, shown in symbolic representation. Anomeric configurations at glycosidic bonds labelled by α and β; attachment positions shown by bond orientation according to the legend. “S” indicates sulfonation at the stated position; “OMe” indicates methylation at the reducing end anomeric hydroxyl group.

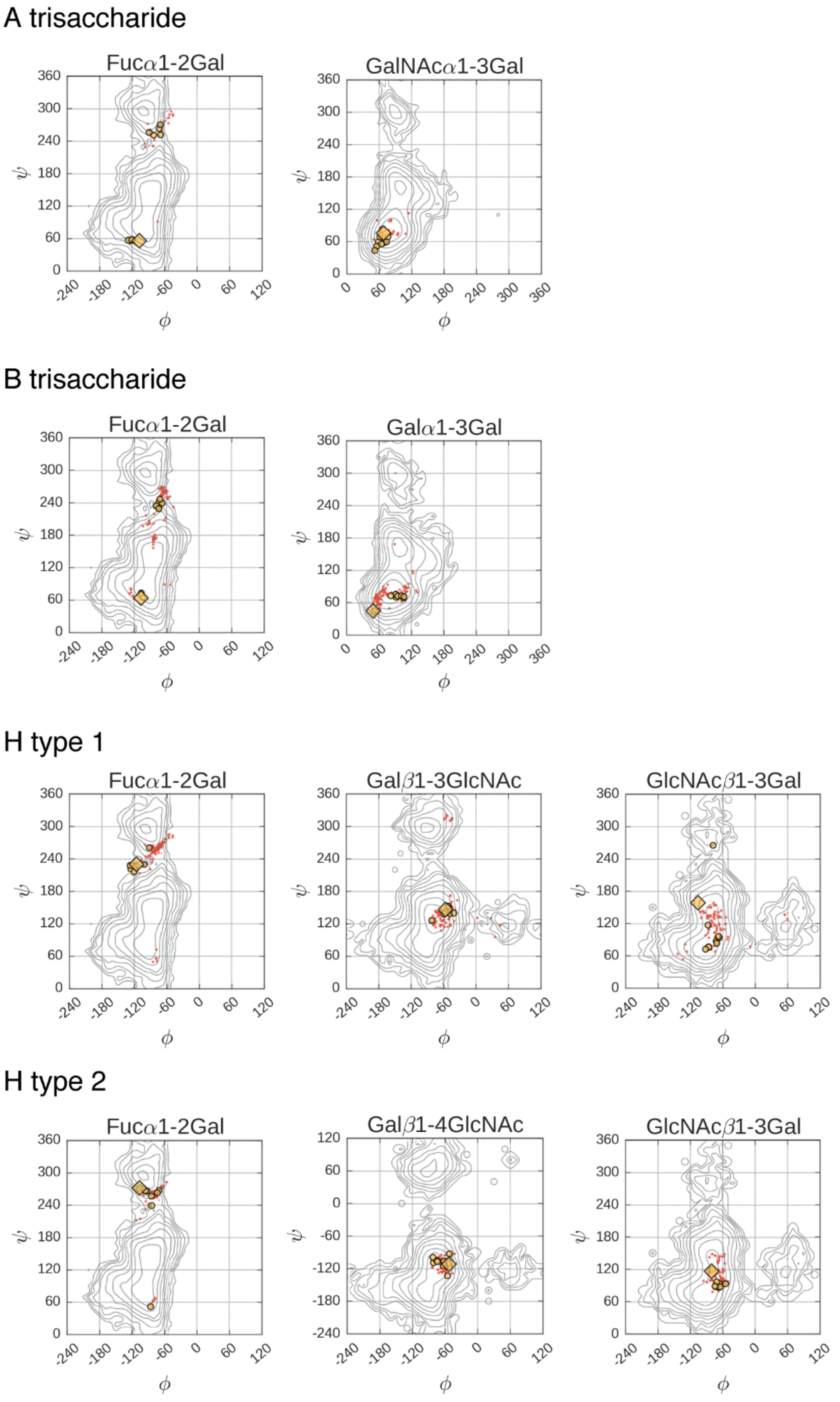

Supplementary Figure 2. Glycosidic torsion angles from docked poses compared to calculated energetic landscapes. Dihedrals defined as: φ, O_5-C_1-O_1-C_x; ψ, C_1-O_1-C_x-C_{x+1}. Contour plots colour coding: grey, calculated energy landscapes of constituting linkages; red, docked pose dihedrals; yellow circles, top ten ranked docked pose dihedrals; yellow diamond, top ranked pose dihedral. Contour plot lines mark intervals of 1 kcal/mol.

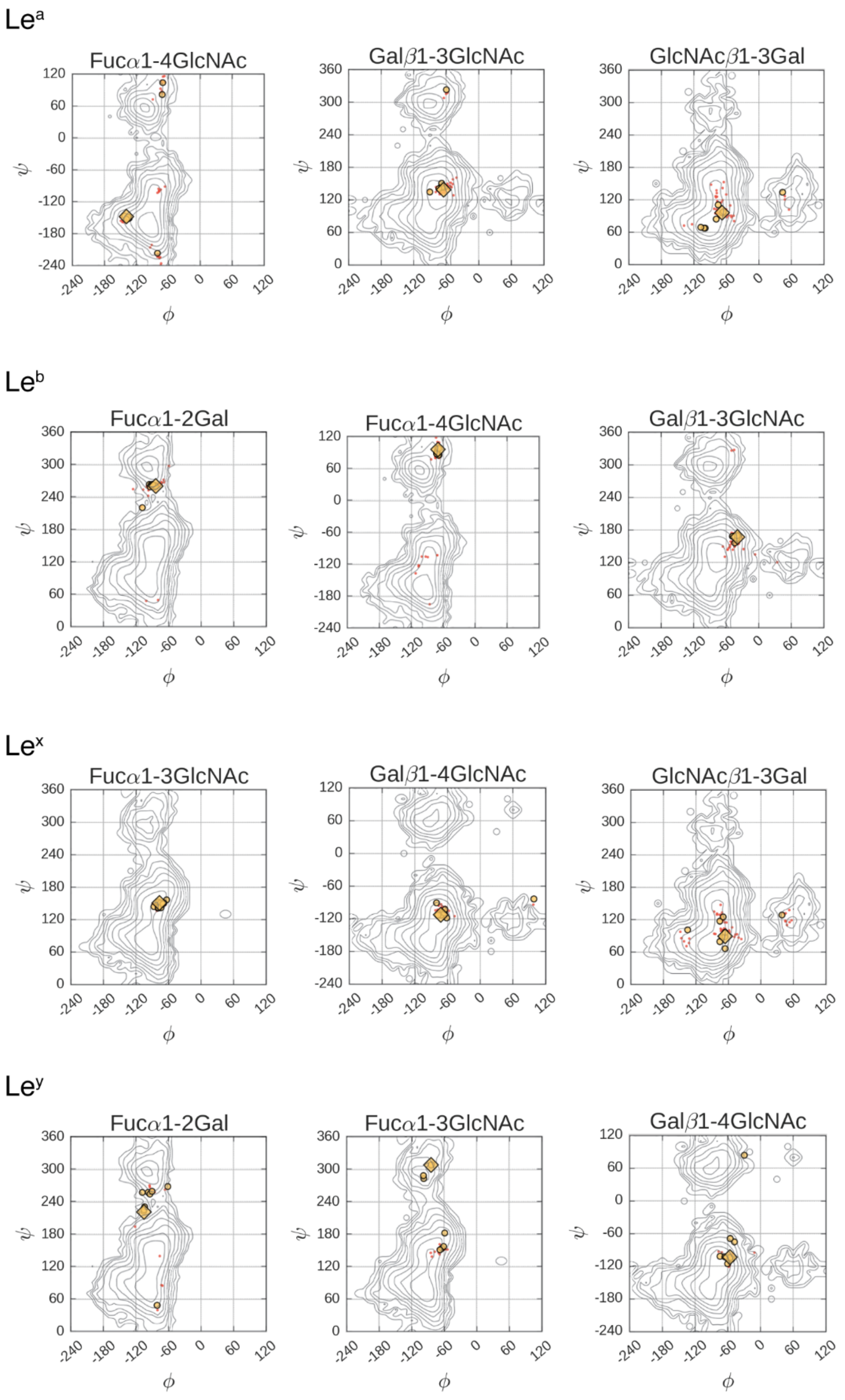

Supplementary Figure 3. Glycosidic torsion angles from docked poses compared to calculated energetic landscapes. Dihedrals defined as: φ, O_5-C_1-O_1-C_x; ψ, C_1-O_1-C_x-C_{x+1}. Contour plots colour coding: grey, calculated energy landscapes of constituting linkages; red, docked pose dihedrals; yellow circles, top ten ranked docked pose dihedrals; yellow diamond, top ranked pose dihedral. Contour plot lines mark intervals of 1 kcal/mol.

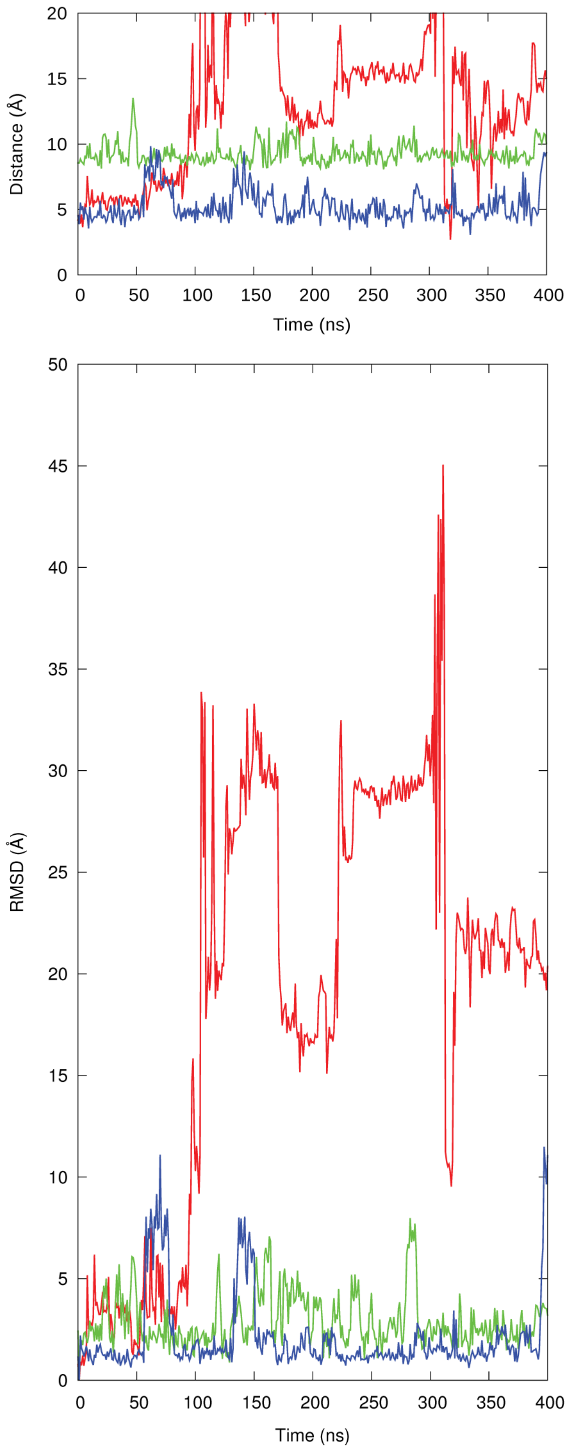

Supplementary Figure 4. Top: distance between docked pose Le^x fucose saccharide and Arg15 sidechain during molecular dynamics simulation. Bottom: Ligand RMSD for all atoms of the docked pose Le^x saccharide during molecular dynamics simulation. Each plot shows all three triplicate simulations.

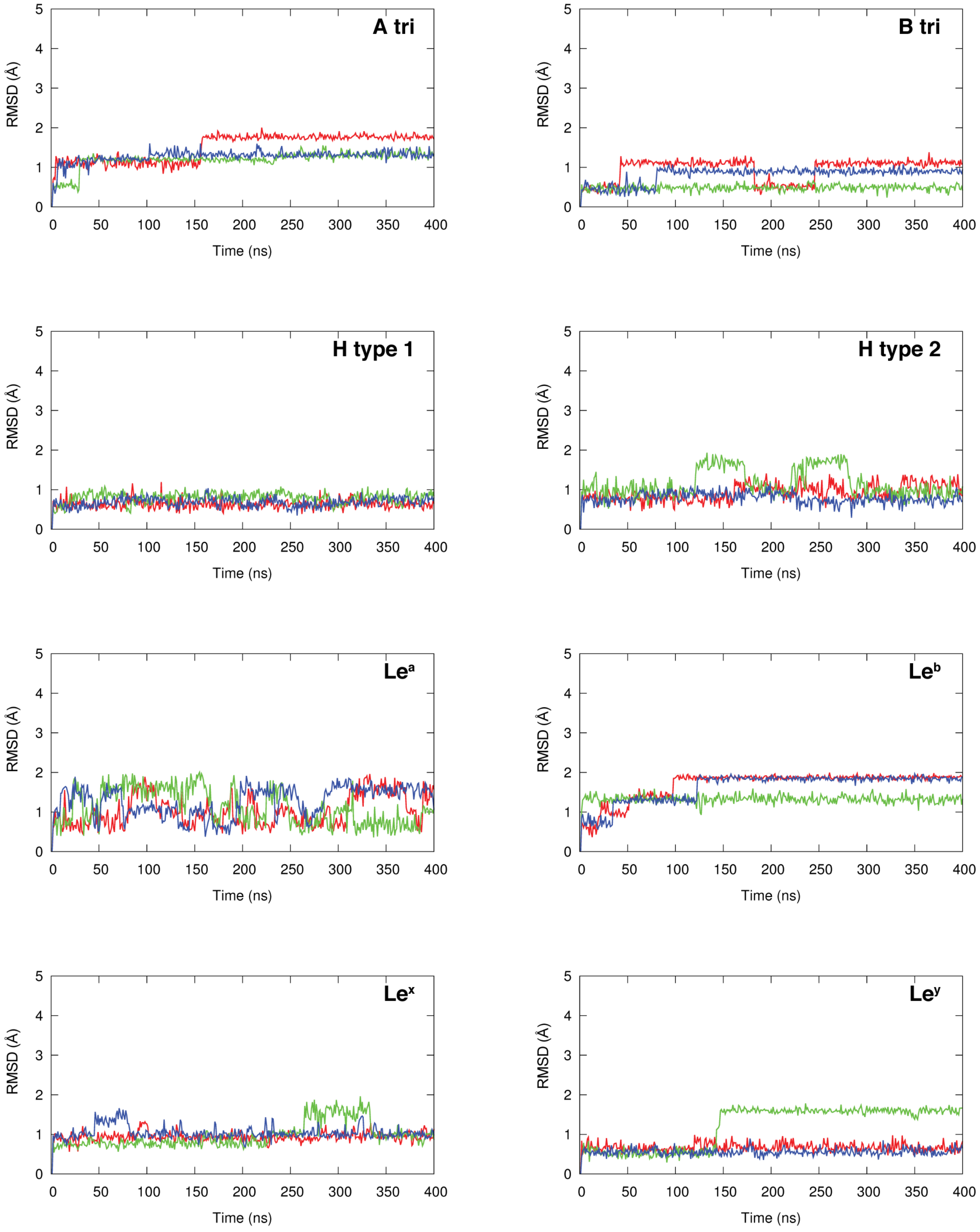

Supplementary Figure 5. Ligand RMSD for all atoms of the blood-group carbohydrates in complex with BambL during MD simulation. All simulation replicates are shown in each plot, coloured in red, blue, and green.

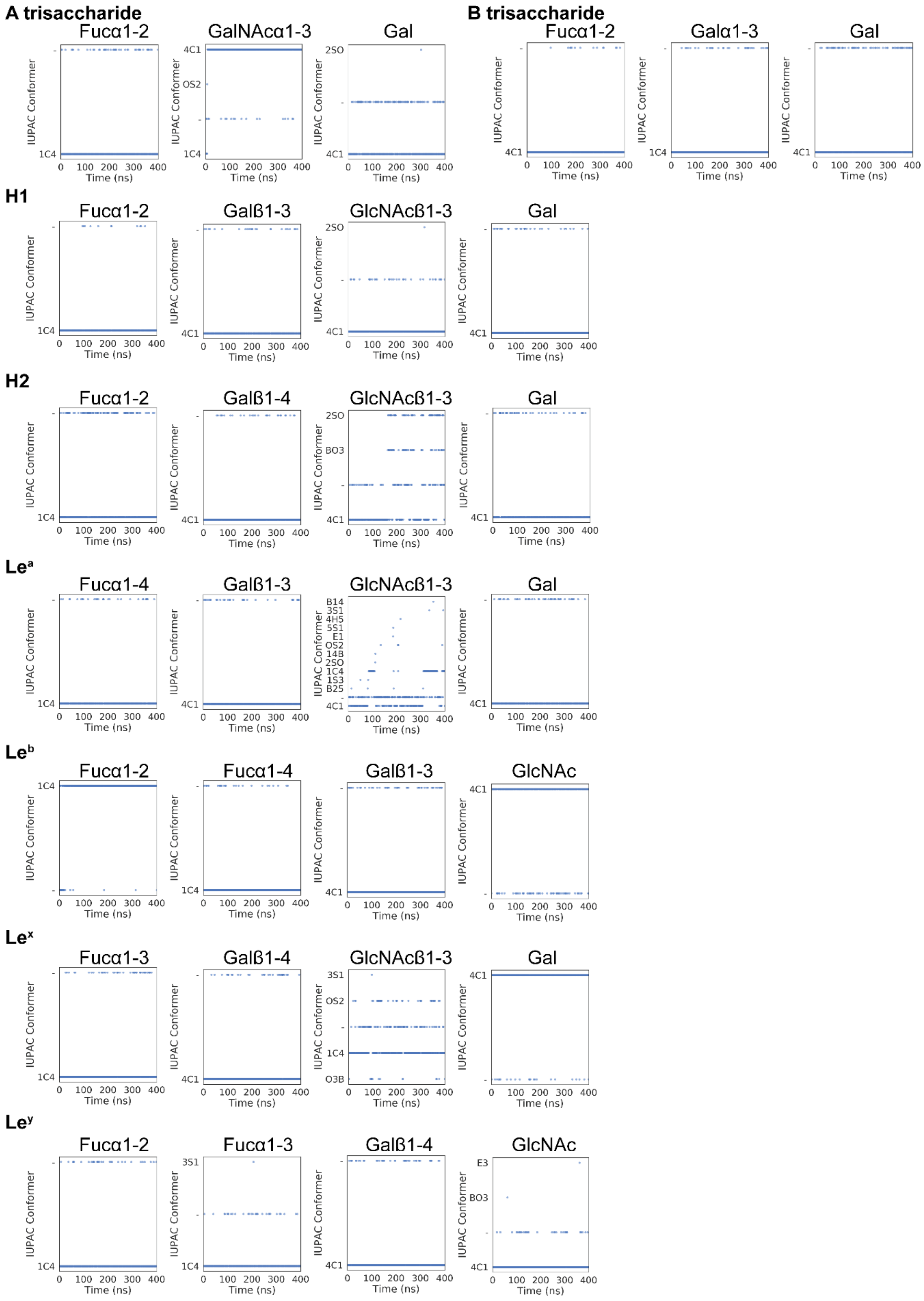

Supplementary Figure 6. Carbohydrate ring conformations for all blood group carbohydrates in complex with BambL during MD simulation. Measurements shown are from a single representative replicate simulation. Conformer labels correspond to IUPAC nomenclature.

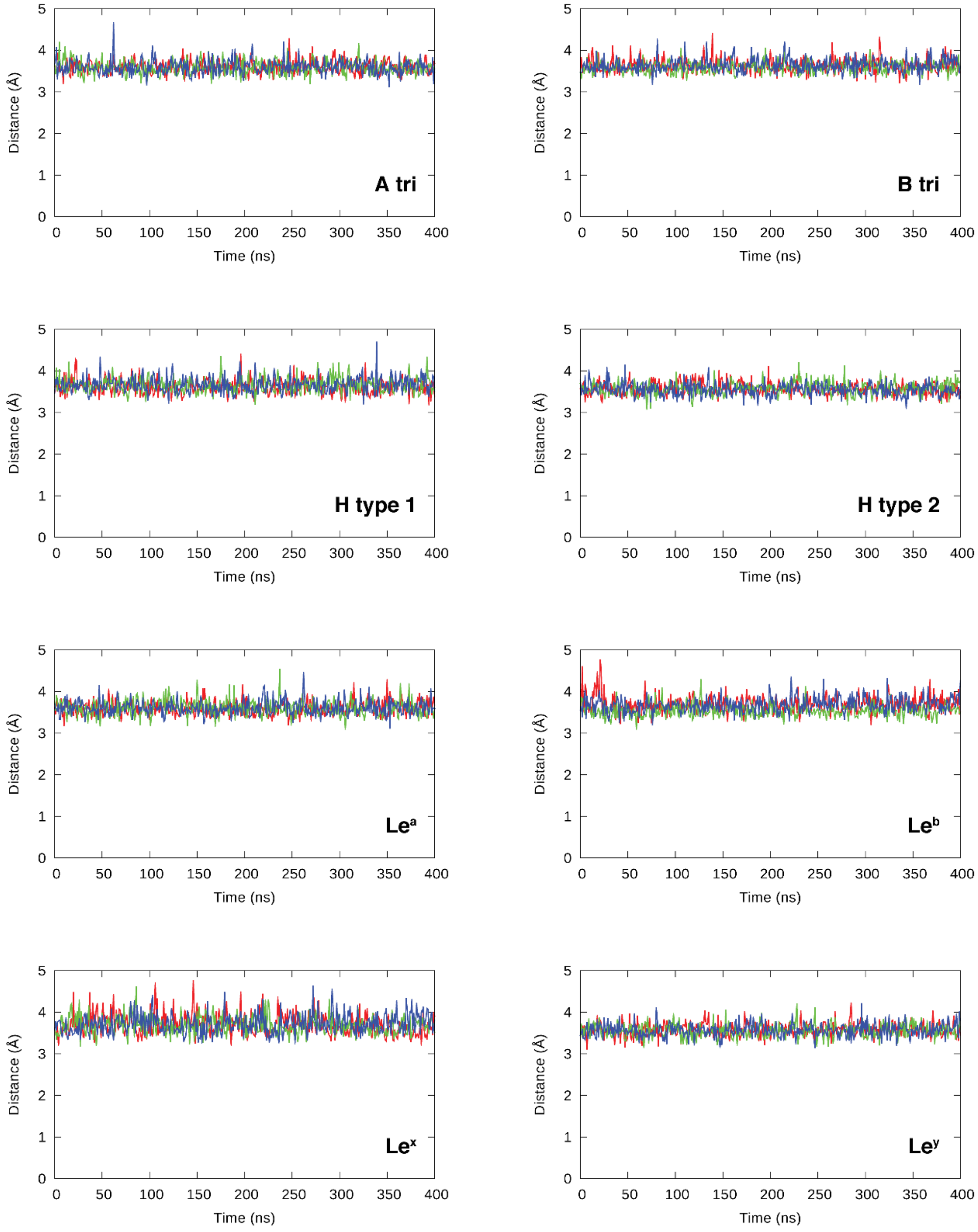

Supplementary Figure 7. Shortest distance between buried fucose saccharide atoms C3, C4, C5, or C6 and Trp74 indole moiety during MD simulation. All simulation replicates are shown, coloured in red, blue, and green.

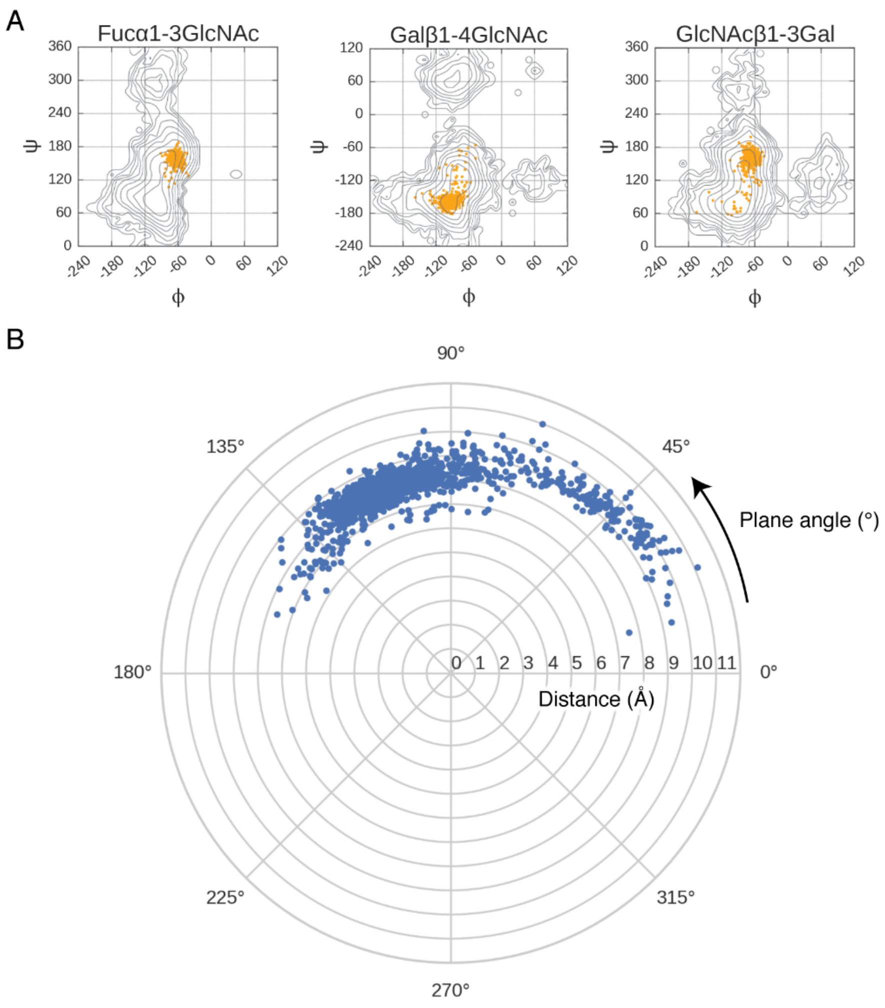

Supplementary Figure 8. Conformations adopted by Le^x saccharide during MD simulation. (A) Glycosidic dihedral angles for each linkage. Dihedrals defined as: φ, O_5-C_1-O_1-C_x; ψ, C_1-O_1-C_x-C_{x+1}. (B) polar coordinate system representation of saccharide shape adapted from Topin, et al. (2016). Radial axis shows the distance between Fuc-C4 and Gal-O4, polar coordinate representing the angle between the fucose and galactose ring planes, each defined by the saccharide O_5-C_2-C_5 atoms.

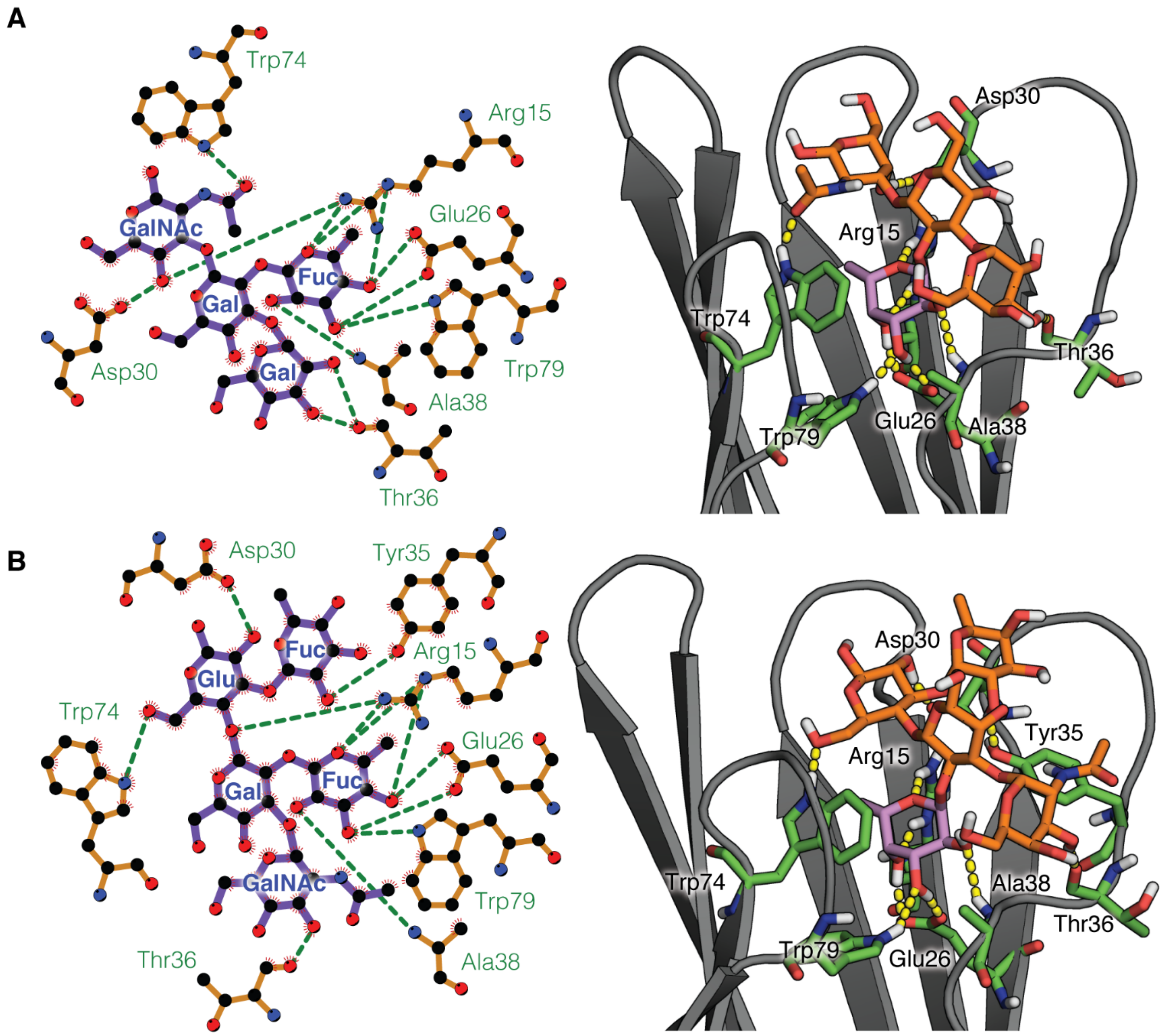

Supplementary Figure 9. Binding modes featuring simultaneously highly occupied hydrogen bonds to fucose and non-fucose saccharides. (A) compound **21**; (B) compound **33**. In LigPlot diagrams: hydrogen bonds shown as green dashed lines, intermolecular van der Waals contacts shown as red spokes radiating towards contacting atoms, ligand bonds shown in purple, protein bonds shown in orange. In 3D structures, ligand shown in orange with buried fucose shown in purple, protein main chain shown as ribbon cartoon. Key residue side chains are shown and labelled. Non-polar hydrogen atoms omitted for clarity.

Table SI. Interactions between the buried fucose saccharide and BambL

Ligand atom(s)	Protein atom	Distance in docked pose (Å)								
		A tri	B tri	H1	H2	Le[a]	Le[b]	Le[y]	Mean	SD
					Hydrogen bonds					
O2	Ala38-H	1.84	1.99	1.86	2.13	2.08	2.13	2.15	2.03	0.1
O3	Trp79-HE1	1.86	2.05	1.80	1.93	2.13	2.07	1.93	1.97	0.1
	Glu26-OE1	2.00	1.67	1.89	1.90	1.78	2.08	1.90	1.89	0.1
	Glu26-OE2	2.64	2.37	2.71	2.84	2.39	2.85	2.84	2.66	0.2
O4	Glu26-OE2	1.99	2.82	1.78	1.79	1.91	2.13	1.77	2.03	0.4
	Arg15-HE	2.13	1.84	2.45	2.33	1.84	2.09	2.35	2.15	0.2
O5	Arg15-HH21	1.83	1.82	1.64	1.89	1.78	1.80	1.88	1.81	0.1
					Hydrophobic stacking					
C3	Trp74 phenyl	4.00	4.27	4.16	4.10	4.16	4.02	4.12	4.12	0.1
C4	Trp74 phenyl	3.91	4.16	3.93	3.86	4.16	3.85	3.86	3.96	0.1
C5	Trp74 pyrrole	3.86	3.88	3.90	3.69	3.89	3.90	3.66	3.83	0.1
C6	Trp74 pyrrole	3.94	3.98	3.70	3.49	4.07	3.78	3.38	3.76	0.3
